# Effect of hyperchloremia on acute kidney injury in critically ill septic patients: a retrospective cohort study

**DOI:** 10.1186/s12882-017-0750-z

**Published:** 2017-12-02

**Authors:** Lenar Yessayan, Javier A. Neyra, Fabrizio Canepa-Escaro, George Vasquez-Rios, Michael Heung, Jerry Yee, for the Acute Kidney Injury in Critical Illness Study Group

**Affiliations:** 1 Division of Nephrology, University of Michigan, 3914 Taubman Center, 1500 E. Medical Center Dr. 5364, Ann Arbor, MI 48109-5364 USA; 2 Division of Nephrology, Bone and Mineral Metabolism, University of Kentucky Medical Center, Lexington, KY USA; 3 Center for Mineral Metabolism and Clinical Research, University of Texas Southwestern, Dallas, TX USA; 4 Division of Hospitalist Medicine, University of Kentucky Medical Center, Lexington, KY USA; 5 Division of Nephrology and Hypertension, Henry Ford Hospital, Detroit, MI USA

**Keywords:** Hyperchloremia, Sepsis, Acute kidney injury, Chloride load

## Abstract

**Background:**

Hyperchloremia is common in critically ill septic patients. The impact of hyperchloremia on the incidence of acute kidney injury (AKI) is not well studied. We investigated the association between hyperchloremia and AKI within the first 72 h of intensive care unit (ICU) admission.

**Methods:**

6490 ICU adult patients admitted with severe sepsis or septic shock were screened for eligibility. Exclusion criteria included: AKI on admission, baseline estimated glomerular filtration rate (eGFR) <15 ml/min/1.73 m^2^, chronic renal replacement therapy, absent baseline serum creatinine data, and absent serum chloride data on ICU admission.

**Results:**

A total of 1045 patients were available for analysis following the implementation of eligibility criteria: 303 (29%) had hyperchloremia (Cl_0_ ≥ 110 mEq/L) on ICU admission, 561 (54%) were normochloremic (Cl_0_ 101–109 mEq/L) and 181 (17%) were hypochloremic (Cl_0_ ≤ 100 mEq/L). AKI within the first 72 h of ICU stay was the dependent variable. Chloride on ICU admission (Cl_0_) and change in Cl by 72 h (ΔCl = Cl_72_ – Cl_0_) were the independent variables. The odds for AKI were not different in the hyperchloremic group when compared to the normochloremic group [adjusted odds ratio (OR) =0.80, 95% confidence interval [CI] (0.51–1.25); *p* = 0.33] after adjusting for demographics, comorbidities, baseline kidney function, drug exposure and critical illness indicators including cumulative fluid balance and base deficit. Furthermore, within the subgroup of patients with hyperchloremia on ICU admission, neither Cl_0_ nor ΔCl was associated with AKI or with moderate/severe AKI (KDIGO Stage ≥2).

**Conclusions:**

Hyperchloremia occurs commonly among critically ill septic patients admitted to the ICU, but does not appear to be associated with an increased risk for AKI within the first 72 h of admission.

## Background

Chloride is the most abundant anion in the extracellular fluid and the second most important contributor to plasma tonicity [1]. It plays an essential role in many body functions including acid-base balance, muscular activity, osmosis, and immunomodulation [2]. The possibility of harm from hyperchloremia, particularly in the context of fluid resuscitation with chloride-liberal solutions, has recently garnered the research interest of the scientific community. Recent observational studies have shown increased mortality with chloride rich solutions, [3] chloride load, [4] and hyperchloremia [5, 6]. Animal and human experiments have also suggested that chloride-rich solutions may have a detrimental effect on renal function [7–11]. Proposed explanations of this association include renal vasoconstriction leading to reduction in renal cortical tissue perfusion and renal interstitial edema leading to intracapsular hypertension [7–9]. However, a recent large cluster randomized trial in a heterogeneous population of patients did not demonstrate a difference in the risk of acute kidney injury (AKI) or mortality among those who received buffered crystalloids compared with 0.9% saline [12]. Criticism of this trial includes the lack of data delineating phenotypic characteristics between the two groups such as serum chloride data, the minimal total exposure to these two types of fluid, and the overall low incidence of AKI in the study population.

Therefore we aimed to investigate the association of hyperchloremia on intensive care unit (ICU) admission and the development of AKI within 72 h in critically ill patients with severe sepsis or septic shock, a group of patients with high risk for AKI. We also aimed to investigate whether AKI incidence varied by admission serum chloride level or by serum chloride level changes in the first 72 h in patients who were hyperchloremic on ICU admission. Our hypothesis was that high chloride levels would be associated with the occurrence of AKI.

## Methods

### Study design and participants

We conducted a single-center, retrospective cohort study utilizing a population-based, ICU database of adult patients with severe sepsis or septic shock admitted to Henry Ford Hospital, an urban, tertiary care hospital from May 2007 through April 2012. Severe sepsis or septic shock was defined by Angus criteria [13], using the International Classification of Diseases, Ninth Revision, Clinical Modification (ICD-9-CM) codes [14] for a bacterial or fungal infection and a diagnosis of acute organ dysfunction excluding gastrointestinal failure. Exclusion criteria included the presence of any of the following: absent serum creatinine (SCr) measurement within 3 months prior to ICU admission; baseline estimated glomerular filtration rate (eGFR) < 15 ml/min/1.73 m^2^ using the 4-variable Modification of Diet in Renal Disease study eq. [15]; patients on chronic renal replacement therapy; patients with AKI or absent serum chloride data on ICU admission; and those with absent recorded daily fluid balance within the first 72 h of ICU stay. Data were electronically extracted from electronic health record (EHR) by data management personnel blinded to the study. The accuracy of data collection was further validated by individual EHR review of 10% of the data. The protocol was approved by the institutional review board of Henry Ford Hospital (IRB #7044).

### Study variables

Serum chloride was measured by indirect potentiometry (SYNCHRON Systems, Beckman Coulter Inc., Brea, CA). The delta chloride (ΔCl) was defined as the difference between serum chloride at 72 h (Cl_72_) and serum chloride on ICU admission (Cl_0_). The Acute Physiology and Chronic Health Evaluation II (APACHE II) and Sequential Organ Failure Assessment (SOFA) scores were calculated after integration of clinical and laboratory data within the first day of ICU admission. Cumulative fluid balance was calculated based on total fluid input minus output within the first 72 h of ICU stay. These data did not include pre-ICU fluid administration. Base deficit was calculated by subtracting the serum HCO_*3*_^−^ measurement on ICU admission from the normal serum HCO_*3*_^−^ value of 24 mEq/L. Subject-specific variables were obtained from EHRs. Comorbidities (e.g., diabetes, hypertension, and heart failure) were identified using ICD-9-CM codes, except for anemia that was defined as admission hematocrit <39% for men and <36% for women. Data pertaining to drug exposure, red blood cell transfusion, and mechanical ventilation were based on hospital billing codes for the indexed admission within the time frame of the study.

### Study outcomes

The primary outcome measure was the occurrence of any AKI (KDIGO Stage ≥1), or moderate/severe AKI (KDIGO Stage ≥2) within the first 72 h of ICU stay and was adjudicated based on Kidney Disease Improving Global Outcomes consensus SCr-based criteria by comparing the highest SCr measured within the first 72 h of ICU admission and the reference SCr within 3 months before admission [16].

### Statistical analysis

The study sample was divided into 3 subgroups based on serum chloride (Cl) levels at the time of ICU admission: hyperchloremia (Cl_0_ ≥ 110 mEq/L), normochloremia (Cl_0_ 101–109 mEq/L) and hypochloremia (Cl_0_ ≤ 100 mEq/L). Categorical data were reported as percentages and continuous data as means ± standard deviation or median (25th – 75th percentile). For categorical variable comparison between the three subgroups, the chi-square test was used. Analysis of variance (ANOVA) was used for continuous variable comparisons when data were normally distributed and the Kruskal-Wallis test was used for non-normally distributed data.

The associations between AKI (any AKI or moderate/severe AKI) within 72 h (dependent variable) and 1) serum chloride subgroups on ICU admission and 2) admission serum chloride levels (Cl_0_) within the hyperchloremic subgroup and 3) delta serum chloride at 72 h (ΔCl = Cl_72_ – Cl_0_) within the hyperchloremic subgroup were examined using multivariable logistic regression models. These associations were also tested within the hypochloremic subgroup. For the three chloride subgroup comparison in the incidence of AKI, a multivariable logistic regression model was used. The model was adjusted for confounders that were unequally distributed between the three subgroups (*p*-value for comparison <0.25 in Table 1). To further test whether worsening hyperchloremia is associated with AKI in the hyperchloremic subgroup, we evaluated the association of serum chloride on ICU admission (Cl_0_) and delta chloride (ΔCl = Cl_72_ – Cl_0_) with the incidence of AKI within 72 h in this subgroup. The multivariable logistic models included all variables with *P*-value of <0.25 in univariate models of AKI. Candidate variables included demographic data (age, gender, and race); comorbidity (baseline eGFR, diabetes, hypertension, heart failure, and anemia); indicators of critical illness (oliguria, APACHE II, SOFA, cumulative fluid balance, base deficit, mechanical ventilation, red blood cell transfusion); and drug exposure (diuretic, statin, aminoglycoside, and intravenous or intra-arterial iodine contrast). Only 1 of 2 variables was included in the event of collinearity between variables. The 95% CIs reported for the logistic regression odds ratios (ORs) were calculated by the Wald estimation. Two-sided *P*-values <0.05 indicated statistical significance. Spreadsheet software and SAS 9.3 (SAS Institute, Cary, NC) were used for data acquisition and analysis.

**Table 1:** Clinical characteristics stratified by 3 serum chloride subgroups at the time of ICU admission: Hyperchloremia (Cl_0_ ≥ 110 mEq/L); Normochloremia, (Cl_0_ 100–109 mEq/L); and Hypochloremia (Cl_0_ ≤ 100 mEq/L)

Variable	Admission Serum Chloride (Cl_0_) ≥110 mEq/L (*n* = 303)	Admission Serum Chloride (Cl_0_) 101–109 mEq/L (*n* = 561)	Admission Serum Chloride (Cl_0_) ≤100 mEq/L (*n* = 181)	*P*-value
*Demographics*				
Age, years, mean ± SD	67.9 ± 15.6	64.8 ± 16.7	67.0 ± 14.6	0.0146*
Male, %	44.9%	53.7%	55.8%	0.0214*
African-American, %	42.6%	33.2%	24.9%	0.0002*
*Chronic conditions*				
Baseline SCr, mg/dl, median (IQR)	1.2 (0.9–1.7)	1.2 (0.9–1.6)	1.2 (0.9–1.6)	0.0794
Baseline eGFR, mL/min/1.73m^2^, median (IQR)	57.0 (40.4–76.8)	63.1 (44.2–87.9)	61.2 (42.1–88.0)	0.1032
Diabetes, %	23.8%	20.1%	21.6%	0.4648
Hypertension, %	46.5%	46.5%	43.7%	0.8050
Heart failure, %	3.3%	2.1%	4.4%	0.2423
Anemia, %	88.6%	85.1%	74.4%	0.0001*
*Drug Exposure*				
Diuretic, %	44.2%	44.0%	55.8%	0.0161*
Statin, %	28.7%	30.8%	30.4%	0.8073
Iodine contrast, %	27.7%	33.9%	29.3%	0.1446
Aminoglycoside, %	8.3%	6.1%	1.7%	0.0119*
*Critical indicators*				
Oliguria, %	9.0%	6.2%	5.3%	0.2467
CFB 72 h, liters, median (IQR)	3.1 (−0.1–8.0)	2.0 (−0.5–5.4)	0.75 (−2.1–3.8)	0.0005*
Pressor or inotrope, %	37.3%	30.1%	27.6%	0.0408*
Mechanical ventilation, %	51.2%	39.4%	33.2%	<0.0001*
Red blood cell transfusion, %	6.6%	2.0%	0.6%	<0.0001*
Base Deficit, mmol/L Median (IQR)	3.6 (1.7–6.4)	1.1 (−1.7–4.0)	−2.6 (−7.4–2.4)	<0.0001
APACHE II score, mean ± SD	14.9 ± 7.0	12.1 ± 5.9	11.80 ± 5.06*	<0.0001
SOFA score, mean ± SD	5.8 ± 3.8	4.5 ± 3.4	4.4 ± 3.4*	<0.0001

*eGFR = estimated glomerular filtration rate based on Modification of Diet in Renal Disease (MDRD) Study equation; SCr = serum creatinine; iodine contrast only if intravenous or intra-arterial; CFB = cumulative fluid balance; oliguria defined as urine output less than 500 ml in 24 h; anemia = admission hematocrit <39% for men and <36% for women; APACHE II = Acute Physiology and Chronic Health Evaluation II; SOFA = Sequential Organ Failure Assessment

## Results

**Fig. 1:**
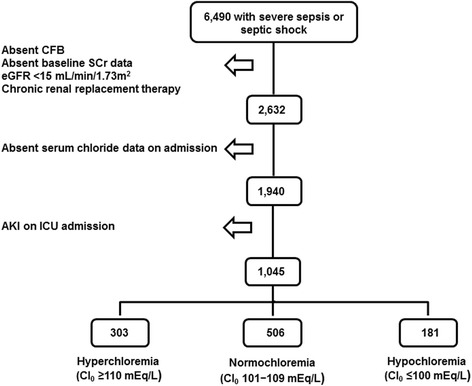
Flowchart of Patient Inclusion and Reasons for Exclusion. CFB = cumulative fluid balance; Cl_0_ = serum chloride at the time of ICU admission; eGFR = estimated glomerular filtration rate; ICU = intensive care unit; SCr = serum creatinine; AKI = Acute kidney injury

Of 6490 patients examined for eligibility, 1045 satisfied inclusion and exclusion criteria (Fig. 1). Age was 66.0 ± 16.1 years, 360 (34.5%) were African Americans, 479 (45.8%) were male. A total of 482 (46.1%) patients were either on vasopressors or inotropes and 315 (30.1%) were on diuretics. The median cumulative fluid balance in the first 72 h of ICU stay was 2063 ml [25th – 75th percentile, −678 to 6029 ml]. The mean SOFA score was 4.9 ± 3.6 and APACHE II score was 12.9 ± 6.2. The mean base deficit was 1.9 ± 7.0 mEq/L. Serum chloride on ICU admission was 106 ± 7 mEq/L (range 82–130 mEq/L) and 237 (22.7%) of patients developed AKI within the first 72 h of ICU stay.

Of the 1045 patients that satisfied eligibility criteria, 303 (29.0%) were hyperchloremic (Cl_0_ ≥ 110 mEq/L) on ICU admission, 561 (53.7%) were normochloremic (Cl_0_ 101–109 mEq/L) and 181 (17.3%) were hypochloremic (Cl_0_ ≤ 100 mEq/L). Baseline characteristics by admission chloride subgroup are shown in Table 1. The following variables were unequally distributed between the three serum chloride subgroups: age, gender, race, anemia, heart failure, aminoglycoside use, diuretic administration, intravenous or intra-arterial iodine exposure, pressor or inotrope use, cumulative fluid balance, % weight gain, mechanical ventilation, red blood cell transfusion, base deficit, oliguria and indices of critical illness severity (APACHE II and SOFA scores). A multivariable logistic regression model adjusted for the aforementioned variables did not show any difference in the odds for any AKI in the hyperchloremic group when compared to the normochloremic group [adjusted odds ratio = 0.80, 95% confidence interval [CI] (0.51–1.25); *p* = 0.33] and lower odds for AKI when compared to the hypochloremic group [adjusted OR = 0.50, 95% CI (0.28–0.91); *p* = 0.03]. The odds for moderate/severe AKI in the hyperchloremic group were not different from the normochloremic group [adjusted odds ratio = 0.97, 95% CI (0.50–1.91); *p* = 0.93] or the hypochloremic group [adjusted OR = 0.42, 95% CI (0.18–1.01); *p* = 0.05].

The independent association of both serum chloride levels on admission (Cl_0_) and delta chloride (ΔCl) with AKI at 72 h was assessed in multivariable models within the hyperchloremic subgroup. All variables with *P* values of <0.25 from the univariate analyses were included in the multivariate analyses. Serum chloride level on admission (Cl_0_) was not associated with the occurrence of AKI at 72 h [adjusted odds ratio per 1 mEq/L increase in Cl_0_ = 0.99, 95% CI (0.89–1.09); *p* = 0.81] or moderate/severe AKI at 72 h [adjusted odds ratio per 1 mEq/L increase in Cl_0_ = 0.95, 95% CI (0.81–1.11); *p* = 0.52] (Table 2). Furthermore, within-subject change in serum chloride (ΔCl) during the first 72 h of ICU stay was also not associated with either AKI at 72 h [adjusted odds ratio per 1 mEq/L increase in ΔCl =1.04, 95% CI (0.97–1.11); *p* = 0.27] or moderate/severe AKI at 72 h [adjusted odds ratio per 1 mEq/L increase in ΔCl =1.03, 95% CI (0.93–1.14); *p* = 0. 0.62] (Table 3).

**Table 2:** Univariable analyses of determinants of any AKI and multivariable analyses investigating the association between chloride levels on ICU admission and 1) any AKI at 72 h and 2) moderate/severe AKI at 72 h

Variable	Any AKI		Any AKI Hyperchloremic Subgroup		Moderate/Severe AKI Hyperchloremic Subgroup	
	Odds Ratio Univariable	*P*-value	Odds Ratio Multivariable	*P*-value	Odds Ratio Multivariable	*P*-value
Cl_0_ per mEq/L increase	1.01 (0.94–1.09)	0.7317	0.99 (0.89–1.09)	0.8072	0.95 (0. 81–1.11)	0.5200
Age, years	1.00 (0.98–1.01)	0.5412	–	–	–	–
Male	1.65 (0.95–2.87)	0.0774	1.72 (0.80–3.69)	0.1625	3.03 (0.89–10.29)	0.0750
African-American	1.25 (0.72–2.17)	0.4338	–	–	–	–
Baseline eGFR per mL/min/1.73m^2^	1.00 (0.99–1.01)	0.9957	–	–	–	–
Diabetes	0.61 (0.30–1.23)	0.1672	0.54 (0.22–1.35)	0.1874	0.75 (0.22–2.61)	0.6514
Hypertension	0.63 (0.36–1.10)	0.1045	0.753 (0.37–1.54)	0.4370	1.02 (0.36–2.93)	0.9655
Heart failure	3.97 (1.11–14.15)	0.0338	3.86 (0.93–16.06)	0.0633	1.90 (0.22–16.62)	0.5640
Anemia	1.26 (0.50–3.18)	0.6305	–	–	–	–
Diuretic	2.37 (1.34–4.16)	0.0028	2.631 (1.21–5.74)	0.0150	1.75 (0.56–5.45)	0.3362
Statin	0.64 (0.33–1.22)	0.1760	0.47 (0.21–1.07)	0.0728	1.00 (0.31–3.22)	0.9996
Iodine contrast	1.24 (0.68–2.27)	0.4783	–	–	–	–
Aminoglycoside	1.20 (0.46–3.14)	0.7131	–	–	–	–
Oliguria	5.35 (2.25–12.72)	0.0002	3.54 (1.20–10.46)	0.0223	10.77 (2.84–40.85)	0.0005
CFB 72 h per 100 ml increase	1.00 (1.00–1.02)	0.0342	1.00 (1.00–1.01)	0.1772	1.05 (1.00–1.01)	0.0910
Pressor or inotrope	2.49 (1.42–4.37)	0.0014	1.28 (0.53–3.05)	0.5839	1.77 (0.45–6.87)	0.4122
Mechanical ventilation	1.96 (1.11–3.46)	0.0212	0.84 (0.34–2.03)	0.6944	0.72 (0.19–2.76)	0.6359
Red blood cell transfusion	0.64 (0.18–2.26)	0.4908	–	–	–	–
Base Deficit per mEq/L increase	1.03 (0.98–1.09)	0.2254	1.06 0.99–1.14	0.1211	1.10 (1.00–1.20)	0.0512
APACHE II score, per unit increase	1.05 (1.01–1.09)	0.0231	–	–	–	–
SOFA score per unit increase	1.19 (1.11–1.29)	<.0001	1.12 (0.99–1.27)	0.0772	1.06 (0.88–1.27)	0.5502

*eGFR = estimated glomerular filtration rate based on Modification of Diet in Renal Disease (MDRD) Study equation; SCr = serum creatinine; iodine contrast only if intravenous or intra-arterial; CFB = cumulative fluid balance; oliguria defined as urine output less than 500 ml in 24 h; anemia = admission hematocrit <39% for men and <36% for women; APACHE II = Acute Physiology and Chronic Health Evaluation II; SOFA = Sequential Organ Failure Assessment. Any AKI, KDIGO Stage ≥1 SCr-based; Moderate/severe AKI, KDIGO Stage ≥2 SCr-based

The univariate logistic regression analyses are investigating 20 variables as potential predictors of AKI. The two multivariate models are investigating the association between chloride levels on admission (Cl_0_) and 1) any AKI at 72 h and 2) moderate/severe AKI at 72 h in the hyperchloremic subgroup. Both models are adjusted for variables with *P*-value <0.25 on univariate analysis. Variables included for confounding adjustment in the final model are gender, diabetes, hypertension, heart failure, diuretics, statins, oliguria, cumulative fluid balance, pressor or inotrope, mechanical ventilation, base deficit and Sequential Organ Failure Assessment (SOFA) score. Acute Physiology and Chronic Health Evaluation II (APACHE II) was not included in either multivariable model because of collinearity with SOFA score

**Table 3:** Multivariable analyses investigating the association between delta chloride (Δ Cl) from admission to 72 h post-admission and 1) any AKI at 72 h and 2) moderate/severe AKI at 72 h

Variable	Any AKI Hyperchloremic Subgroup		Moderate/Severe AKI Hyperchloremic Subgroup	
	Odds Ratio Multivariable	*P*-value	Odds Ratio Multivariable	*P*-value
**ΔCl** per mEq/L increase	1.04 (0.97–1.11)	0.2652	1.03 (0.93–1.14)	0.6164
Male	1.49 (0.60–3.70)	0.3948	6.38 (1.16–34.99)	0.0329
Diabetes	0.55 (0.20–1.50)	0.2449	0.88 (0.21–3.73)	0.8645
Hypertension	0.60 (0.26–1.36)	0.2196	0.55 (0.15–1.95)	0.3545
Heart failure	7.06 (1.33–37.52)	0.0219	2.54 (0.15–43.62)	0.5205
Diuretic	3.35 (1.26–8.91)	0.0156	3.61 (0.74–17.60)	0.1118
Statin	0.51 (0.20–1.30)	0.1564	1.12 (0.26–4.83)	0.8795
Oliguria	6.09 (1.66–22.39)	0.0066	33.05 (4.89–223.45)	0.0003
CFB 72 h per 100 ml increase	1.00 (1.00–1.01)	0.4866	1.01 (1.00–1.02)	0.1821
Pressor or inotrope	1.37 (0.51–3.69)	0.5277	1.66 (0.35–8.01)	0.5255
Mechanical ventilation	1.10 (0.36–3.32)	0.8734	1.46 (0.26–8.37)	0.6703
Base Deficit per mEq/L increase	1.05 (0.96–1.15)	0.2777	1.12 (0.96–1.30)	0.1411
SOFA score per unit increase	1.09 (0.95–1.25)	0.2340	0.98 (0.80–1.20)	0.8472

CFB = cumulative fluid balance; oliguria defined as urine output less than 500 ml in 24 h; anemia = admission hematocrit <39% for men and <36% for women;; SOFA = Sequential Organ Failure Assessment; Any AKI, KDIGO Stage ≥1 SCr-based; Moderate/severe AKI, KDIGO Stage ≥2 SCr-based

The two multivariate models are investigating the association between delta chloride at 72 h (ΔCl = Cl_72_ – Cl_0_) and 1) any AKI at 72 h and 2) moderate/severe AKI at 72 h in the hyperchloremic subgroup. Both models are adjusted for variables with P-value <0.25 on univariate analysis reported in Table 2

In the hypochloremic subgroup, and using the same multivariable models, there was no independent association between the occurrence of AKI at 72 h and either serum chloride levels on admission (Cl_0_) [adjusted odds ratio per 1 mEq/L decrease in Cl_0_ = 1.02, 95% CI (0.91–1.15); *p* = 0.67] or delta chloride (ΔCl) [adjusted odds ratio per 1 mEq/L decrease in ΔCl =1.18, 95% CI (0.41–3.40); *p* = 0.66].

## Discussion

In this large retrospective cohort study of critically ill septic patients, we did not find an association between admission hyperchloremia (Cl_0_ ≥ 110 mEq/L) and AKI within 72 h of ICU stay. We also could not detect an association between higher serum chloride levels on admission or worsening serum chloride levels in the first 72 h and AKI among those patients with hyperchloremia at the time of ICU admission. The latter finding further supports the absence of any appreciable detrimental effect of hyperchloremia on kidney function in patients with severe sepsis or septic shock.

Hyperchloremia is prevalent in the ICU, afflicting nearly up to a third of the ICU population in some reports [6, 17]. Over the past few years there has been an increasing focus on the potential impact of chloride load in resuscitation fluid and serum chloride on outcomes in critically ill patients. Several large observational studies have associated chloride-rich crystalloid solutions, chloride load and hyperchloremia with increased hospital mortality and/or with AKI [3–6, 10, 11, 17, 18].

In a large retrospective study, Sen et al. found an association between total chloride load and all-cause mortality in 4710 critically ill non-surgical patients who received at least 60 mL/kg fluid resuscitation within a 24 h period [18]. However, this association did not persist after adjusting for age, volume of administered fluid and baseline severity of illness. Shaw et al. conducted a retrospective study to examine the effect of chloride load among 109,836 patients with systemic inflammatory response and demonstrated an association between higher intravenous chloride loads and hospital mortality [4]. Raghunathan et al. examined non-surgical critically ill patients with sepsis and, using propensity-matching (*n* = 6730), reported an association between the use of chloride-rich solutions and increased risk for hospital mortality but not AKI [3]. We previously showed an association between worsening hyperchloremia and hospital mortality in critically ill septic patients admitted with hyperchloremia [6]. Similarly, a single center prospective study by Boniatti et al. showed that hyperchloremia was associated with hospital mortality. Although serum chloride was not a good predictor of hospital mortality, a clinical model that included albumin, age, SOFA score, in addition to serum chloride provided a good discriminatory ability to predict mortality (area under the receiver operating curve for this model was 0.80 [95% confidence interval, 0.73–0.87]) [5]. Zhang et al. showed that patients with AKI have higher maximum chloride levels than patients who did not develop AKI in a group of medical, surgical and post-cardiac surgery patients. A distinctive difference with our study is that they did not adjust for disease severity scores such as SOFA or APACHE-II, and the serum chloride levels (predictor) were not examined at contemporaneous time points in reference to the occurrence of AKI [11]. We selected AKI within 72 h of ICU admission as the main outcome of our study to adjust for critical clinical parameters during this time period (i.e., SOFA score and cumulative fluid balance) that may confound the relationship between serum chloride levels and AKI.

Proposed explanations of renal damage by chloride-rich solutions include dysregulated tubuloglomerular feedback activated by chloride reaching the macula densa and causing renal afferent arteriole vasoconstriction leading to a reduction in renal cortical tissue perfusion and consequent tissue ischemia. An alternative mechanism is through fluid overload and renal interstitial edema leading to intracapsular hypertension or vasomotor nephropathy [7, 9, 19].

Observational studies have shown an association between chloride- rich solutions and AKI. Yunos and colleagues, in a large prospective study of quasi-experimental design (1533 patients), reported a lower incidence of AKI when a chloride-restrictive fluid strategy was implemented in the ICU (8.4% versus 14.0%, *p* < 0.001) [10]. A distinctive difference with our study is that they did not adjust for disease severity scores such as SOFA or APACHE-II, and the serum chloride levels (predictor) were not examined at contemporaneous time points in reference to the occurrence of AKI [11]. We selected AKI within 72 h of ICU admission as the main outcome of our study to adjust for critical clinical parameters during this time period (i.e., SOFA score and cumulative fluid balance) that may confound the relationship between serum chloride levels and AKI. A recent meta-analysis of 21 studies (6253 patients) found that administration of chloride-rich fluids was associated with increased risk for hyperchloremic acidosis and AKI but not mortality [20].

The association of chloride-rich solutions with AKI was not observed in two recent controlled trials. The SPLIT trial, a major trial of buffered crystalloids versus 0.9% saline among 2278 ICU patients, found no difference in the incidence of AKI or rates of renal replacement therapy between the two strategies [12]. Similarly, the LICRA pragmatic trial, a study comparing perioperative chloride-poor intravenous fluids to chloride-rich intravenous fluids among 1136 patients, did not show a difference in the incidence of AKI after adult cardiothoracic surgery [21]. Similar to the SPLIT trial, our study was conducted in ICU patients and our results are consistent with the SPLIT trial and address some of the reported concerns with the results of the former study, namely the low risk for AKI in their study population (about 9%), the lack of serum chloride data and the small overall total fluid that was administered. In our cohort, the incidence of AKI was 22.7% and serum chloride data on admission was available in 1045 patients. The high frequency of events in our large cohort allowed for multiple adjustments for confounders without overfitting the models.

An inherent limitation to the observational studies linking a biochemical parameter to AKI is the incapability to entirely control for residual confounding. Beyond the observed associations, could there be a biologic explanation linking chloride administration or hyperchloremia to AKI? Augmented pro-inflammatory response [22] and diminished coagulation ability [23] have been observed in hyperchloremic metabolic acidosis and renal vasoconstriction may exist with higher chloride load or hyperchloremia but the clinical significance of these findings as a cause of AKI is uncertain.

The strengths of our study are its large sample size; the careful selection of a representative sample of patients with severe sepsis or septic shock admitted to the ICU; and the multivariable adjustment for clinical confounders directly linked to AKI including cumulative fluid balance and comprehensive critical illness severity scores. Limitations of our study include the absence of data pertaining to the amount of fluid administered before ICU admission, the lack of data pertaining to the type of fluid and the lack of timed urine output measurements for AKI diagnosis. Our study population only included patients with severe sepsis or septic shock with an overall high risk to develop AKI. Our findings do not preclude the possibility of harmful renal effects with higher chloride load or hyperchloremia in other populations or in higher-risk groups. Furthermore, because our analytical horizon was only for 72 h, we cannot completely rule out the possibility of delayed harmful renal effects from hyperchloremia.

## Conclusions

In conclusion, our study did not show an association between hyperchloremia and the incidence of AKI within the first 72 h of ICU stay in critically ill septic patients. It is conceivable that the administration of chloride rich solutions itself, rather than any resulting hyperchloremia, is what confers the risk of AKI. It is also plausible that there is no causal relationship between hyperchloremia or exposure to high chloride solutions and AKI in this population. In the absence of clinical trials showing definite evidence of lack of harm with higher chloride loads, it is probably prudent to minimize chloride load when large amounts of crystalloids have already been administered or when patients are already hyperchloremic. Future randomized control trials evaluating the effect of buffered solutions on AKI should include data pertaining to serum chloride levels during the study period.

## Abbreviations

AKI: Acute kidney injury
APACHE II: Acute Physiology and Chronic Health Evaluation II
CFB: Cumulative fluid balance
eGFR: estimated glomerular filtration rate based on Modification of Diet in Renal Disease (MDRD) Study equation
EHR: Electronic health record
ICD-9-CM: International Classification of Diseases, Ninth Revision, Clinical Modification Codes
ICU: Intensive care unit
KDIGO: Kidney Disease Improving Global Outcomes
OR: Odds ratio
SCr: Serum creatinine
SOFA: Sequential Organ Failure Assessment
